# Screen for Localized Proteins in *Caulobacter crescentus*

**DOI:** 10.1371/journal.pone.0001756

**Published:** 2008-03-12

**Authors:** Jay H. Russell, Kenneth C. Keiler

**Affiliations:** Department of Biochemistry and Molecular Biology, The Pennsylvania State University, University Park, Pennsylvania, United States of America; Baylor College of Medicine, United States of America

## Abstract

Precise localization of individual proteins is required for processes such as motility, chemotaxis, cell-cycle progression, and cell division in bacteria, but the number of proteins that are localized in bacterial species is not known. A screen based on transposon mutagenesis and fluorescence activated cell sorting was devised to identify large numbers of localized proteins, and employed in *Caulobacter crescentus.* From a sample of the clones isolated in the screen, eleven proteins with no previously characterized localization in *C. crescentus* were identified, including six hypothetical proteins. The localized hypothetical proteins included one protein that was localized in a helix-like structure, and two proteins for which the localization changed as a function of the cell cycle, suggesting that complex three-dimensional patterns and cell cycle-dependent localization are likely to be common in bacteria. Other mutants produced localized fusion proteins even though the transposon has inserted near the 5′ end of a gene, demonstrating that short peptides can contain sufficient information to localize bacterial proteins. The screen described here could be used in most bacterial species.

## Introduction

A fundamental regulatory challenge for all cells is to make the correct amount of protein at the proper time, and place it at a precise location. Several proteins in bacteria have been shown to be localized in an area measured in the tens of nanometers and not merely to domains such as the cytoplasm, periplasm, or membrane [1]–[3]. Critical processes such as cell division and differentiation depend on correct protein localization [4], [5], yet for most localized proteins the mechanisms responsible for localization are unknown. In some cases, such as chemoreceptor complexes [6], extensive protein-protein interactions are required for localization. Localization information can also be encoded in relatively short peptide sequences, or locons, that have been shown to direct localization of exogenous proteins [7]. Several protein filaments similar to elements of the eukaryotic cytoskeleton have been discovered in bacteria [8], but it is not yet clear if these filaments play a role in localization of most bacterial proteins. To understand the importance of these various mechanisms and to gauge the degree of order within a bacterial cell, it is necessary to identify as many localized proteins as possible.

One approach to identify localized proteins is to clone and express each gene as a fusion to a reporter such as *gfp*, and observe each clone. When this approach was used in *E. coli*, approximately 20% of the proteins were localized [9]. We have devised another approach, using a transposon to insert *gfp* at arbitrary sites in the genome. The use of transposon mutagenesis greatly decreases the resources required for examining a large number of genes. In addition, because GFP fusions are made at many sites within each gene, it is possible to identify short peptides that contain localization information and uncover localization determinants that are masked in a full-length protein. In this study, transposon mutagenesis was used to identify localized proteins and short localization sequences in the model bacterial system *Caulobacter crescentus*.

*C. crescentus* has an asymmetric morphology that facilitates identification of specific subcellular locations [2]. Cell division in *C. crescentus* produces two different daughter cells, a stalked cell, with a prosthesis at the old pole, and a swarmer cell with a single flagellum at the old pole. Swarmer cells must differentiate into stalked cells before they can replicate their DNA and divide. The stalk and flagellum can be used as markers for the poles of the cell, so that the newer pole can be distinguished from the older pole. Another significant advantage of *C. crescentus* is the ability to study changes in localization in response to development and cell cycle progression. *C. crescentus* swarmer cells can be isolated in a density gradient and these cells will pass synchronously through the cell cycle and developmental program [10]. Synchronized cultures have been used to detect cell-cycle dependent localization of several structural and signaling proteins [2], [11], but the number of proteins whose localization changes through the cell cycle is not known.

## Methods

### Plasmids and Strains

The wild-type *C. crescentus* strain was NA1000 [10]. *C. crescentus* cells were grown in PYE medium or M2G medium at 30°C as previously described [12]. Synchronized cultures of *C. crescentus* were obtained by isolating swarmer cells from a Ludox density gradient [10]. In-frame deletions of *CC0572*, *CC2233*, and *CC3691* were generated using plasmid pNPTS138 with the *sacB* counterselection method [13] as previously described [14].

The mini-Tn5-GFP transposon was constructed by cloning the *egfp* gene from plasmid pEGFP-N2 (Novartis) into the Not I site of mini-Tn5 *Km2* in pUT [15]. This plasmid was mobilized into *C. crescentus* by electroporation [12]. To generate M2-tagged proteins, the pM2C plasmid was constructed by cloning sequence encoding the M2 epitope [16] downstream of the xylose-inducible promoter [17] in pML80 (M. Laub, unpublished). The coding sequence for *CC0572*, *CC3691*, or *ahpC* was then inserted between the promoter and the M2 sequence in pM2C. Production of an M2 fusion protein was induced in *C. crescentus* cells containing a pM2C-derived plasmid by addition of xylose to 0.3%. To produce full-length proteins fused to GFP, the coding sequence for CheR or CC2233 was inserted upstream of the *egfp* gene in pMT426 [18] under control of the vanillate-inducible promoter [19] and expression was induced in cells bearing the plasmid by addition of vanillate to 0.5 mM.

### Localization screen

*C. crescentus* cells were mutagenized by introduction of mini-Tn5-GFP and grown on PYE agar medium with 5 µg/ml kanamycin to select for mutants. Colonies (∼20,000) were scraped into PYE medium, grown at 30°C for one hour, and cells with fluorescence above background levels were isolated by FACS (Stanford Shared FACS Facility) and grown on PYE agar medium. To screen for localized fluorescence signal, cells from individual colonies were grown in liquid medium and observed on wet mounts by epifluorescence microscopy.

To identify transposon insertion sites, mutant genomic DNA was isolated (Purgene) and fragments adjacent to *egfp* were cloned by arbitrary PCR [20] with a specific primer within the *egfp* gene, ArbC (GGCCACGCGTCGACTAGTAC) and arbitrary primers Arb1 (GGCCACGCGTCGACTAGTACNNNNNNNNNNGAT) and Arb6 (GGCCACGCGTCGACTAGTACNNNNNNNNNNACGCC). Alternatively, for inverse PCR [21], genomic DNA was digested with Not I, incubated with T4 ligase at 16°C overnight, and amplified by PCR using primers GFP644Fwd (GAGAAGCGCGATCACATGGTC) and GFP133Rev (GGGTCAGCTTGCCGTAGGTGGCATCGC). Amplified DNA was cloned into pGEM T-Easy (Promega) and sequenced.

### Microscopy

Cells were immobilized on a 1% agar pad and imaged using an Eclipse E600 microscope (Nikon) with a 100× Plan Fluor oil N. A. 1.3 objective in conjunction with a CoolSNAP *fx* CCD camera (Photometrics) controlled by Image-Pro Discovery software (Media Cybernetics). Single microscopy images were deconvolved using the 2D Blind Deconvolution algorithm in AutoQuant software (Media Cybernetics).

For optical sectioning, an IX70 laser scanning confocal microscope (Olympus) with 100× UPlanFL oil N. A. 1.3 objective and Fluoview software was used to obtain a Z-series of confocal images at increments of 0.15 µM. Each Z-series was deconvolved and reconstituted using AutoQuant software.

For immunofluorescence microscopy, cells producing an M2 fusion protein were harvested at OD_660_ 0.3–0.4 and prepared as previously described [6]. Cells were stained with DAPI and probed with anti-FLAG M2-Cy3 antibody (Sigma), or anti-FLAG antibody (Sigma) followed by Alexa Fluor 488 goat anti–mouse IgG (Invitrogen). Images were obtained using an Eclipse 90i microscope (Nikon) with a 60× TIRF N. A. 1.4 objective with a Nikon CoolSNAP HQ CCD camera controlled by Simple PCi (Compix, Inc.).

To quantify the prevalence of localization patterns, at least 3 independent microscopy fields were chosen at random and intact cells were identified by DIC (for epifluorescence) or DAPI staining (for immunofluorescence). The signal in at least 80 intact cells was scored and the results were reported as a percentage of intact cells

## Results

### Transposon Mutagenesis to Identify Localized Proteins

To identify proteins in *C. crescentus* that are localized, the mini-Tn5-GFP transposon was engineered to produce fusions of chromosomally-encoded proteins to GFP. A copy of the *egfp* gene was cloned near the O sequence of the mini-Tn5 *Km2* transposon [15], such that recombination of the transposon in the correct reading frame within a gene would result in a C-terminal fusion of the encoded protein to a 13 amino acid linker followed by GFP. Mutagenesis of *C. crescentus* with mini-Tn5-GFP produced approximately 20,000 kanamycin-resistant colonies.

Cells without GFP were expected to comprise about 90% of the colonies, because they could be produced from integration of the mini-Tn5-GFP in a non-coding region, out of the correct reading frame, or in a gene that was not expressed at high enough levels to produce observable GFP signal. Therefore, before examining cells for localized fusion proteins, clones that did not produce observable amounts of a GFP fusion protein were eliminated. Mutant colonies were pooled and cells producing GFP signal above background were isolated by fluorescence activated cell sorting (FACS). Approximately 2×10^5^ cells were sorted, and 6,100 cells producing detectable GFP signal were isolated and grown on agar medium.

To identify clones with localized fluorescence, cells from each colony were grown to exponential phase in liquid medium and observed using epifluorescence microscopy. The majority of the clones produced an even distribution of cytoplasmic fluorescence, similar to *C. crescentus* producing GFP with no fusion (not shown). However, over 1,000 clones with localized GFP fluorescence were recovered. Twenty-four clones with representative localization patterns were chosen for further investigation, and the transposon insertion site was determined by either arbitrary PCR or inverse PCR, followed by DNA sequencing. The identity of each GFP fusion protein was determined by conceptual translation of the DNA sequence. Of the sequenced clones, seven independent insertions were recovered in each of two genes, *rsaA* and *serA* and one insertion was recovered in each of ten different genes (Table 1).

**Table 1:** Localized GFP fusion proteins.

Protein (function)	Fusion sites (full length) 1	Localization
CheR (protein methyltransferase)	267 (293)	1 focus: pole or mid-cell
AhpC (alkyl hydroperoxide reductase)	36 (187)	1 focus: stalked pole or mid-cell
SerA (serine biosynthesis)	136/187/250/483/491/492/508 (526)	1 focus: stalked pole or mid-cell
Ald (alanine dehydrogenase)	286 (370)	1 focus: stalked pole or mid-cell
RsaA (S-layer protein)	99/171/426/462/541/809/940 (1073)	one pole or both
DnaK (protein chaperone)	458 (631)	2 foci: both poles
CC3691 (conserved hypothetical)	28 (152)	2 foci: stalked pole & mid-cell
CoxA (cytochrome *c* oxidase)	534 (552)	periphery
CC2362 (conserved hypothetical)	192 (252)	periphery
CC3385 (conserved hypothetical)	250 (255)	periphery
CC2233 (hypothetical)	62 (147)	periphery/division plane
CC0572 (conserved hypothetical)	314 (527)	helix-like

1 Last residue of protein before mini-Tn5-GFP.

**Figure 1:**
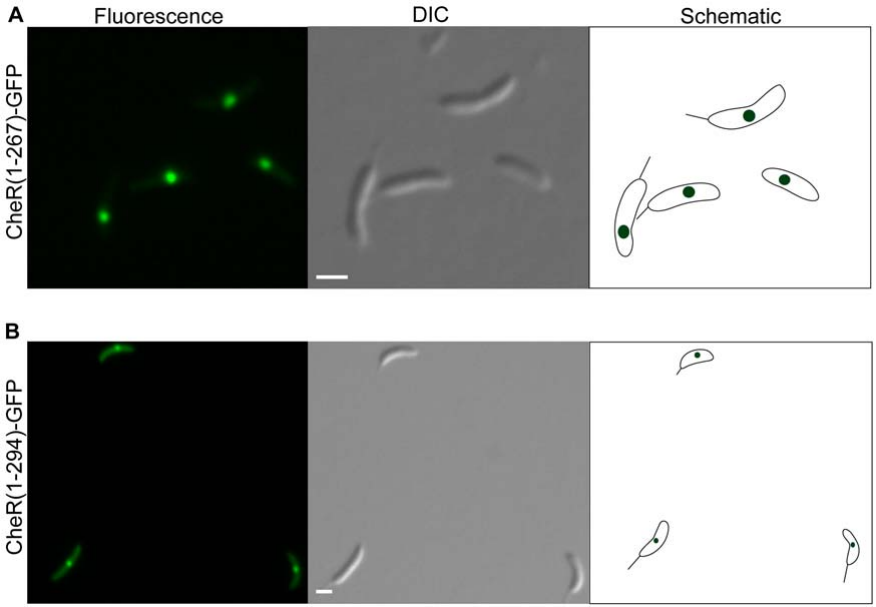
Localization of CheR. *C. crescentus* producing CheR(1–267)-GFP generated by insertion of mini-Tn5-GFP (A) or full-length CheR-GFP from a low copy-number plasmid (B) were imaged by epifluorescence (left panels) and differential interference contrast (DIC) microscopy (center panels). Schematic diagrams (right panels) indicate location of fluorescence signal within the cell. Quantification of images like those shown in panel A indicated 47% of cells had a focus at the pole and 40% had a focus at mid-cell (n = 100). Scale bars indicating 1 µm are shown.

The transposon screen identified five proteins predicted to be localized based on similarity to proteins from other species, or on bioinformatic algorithms. CheR, a protein methyl transferase component of the chemosensory system, was found in a single focus at either the pole (47%) or mid-cell (40%) (Fig. 1A). Although CheR has not been studied in *C. crescentus*, it is localized in a focus near the cell pole in other bacteria [22], [23], and other chemosensory proteins are localized to the flagellar pole in *C. crescentus* [24]. The transposon insertion in *cheR* resulted in fusion of the first 267 residues of the 293 residue protein to GFP. To ensure that the observed localization of CheR was not due to truncation of the protein, the full-length *cheR* gene was cloned as a fusion with *gfp* and expressed from a vanillate-inducible promoter. The full-length CheR-GFP localized with the same pattern observed in the transposon-generated mutant (Fig. 1B). SerA and DnaK fusions also have localization patterns in *C. crescentus* similar to those observed in *E. coli* [9]. SerA, which catalyzes the first reaction dedicated to serine biosynthesis, was localized to a focus that was frequently at the stalked pole or near mid-cell. DnaK, a protein chaperone, was found concentrated at both cell poles (Table 1). Two proteins, CoxA and CC2362, were found around the periphery of the cell. CoxA (cytochrome *c* oxidase) is an integral membrane protein, and is localized to the cell periphery in *E. coli* [9]. CC2362 is predicted to be a peripheral membrane protein (22), and the transposon-generated fusion confirms this prediction. These data indicate that the transposon mutagenesis strategy is capable of identifying proteins expected to be localized.

Seven independent fusions to RsaA, the S-layer protein, were localized to a single focus at the cell pole (Table 1). These results were unexpected, because RsaA is secreted by a dedicated type I transporter, and the mature form of the protein is extracellular [25]. However, the polar localization of C-terminal GFP fusions was not inconsistent with previous studies of RsaA transport. C-terminal truncations of RsaA are not exported because the extreme C terminus contains the export signal, but it has been proposed that the N-terminal region of the protein contains information to target RsaA to the transporter sites [26]. The localization of RsaA-GFP to the cell poles may indicate that the transporter complex is located at the cell poles.

**Figure 2:**
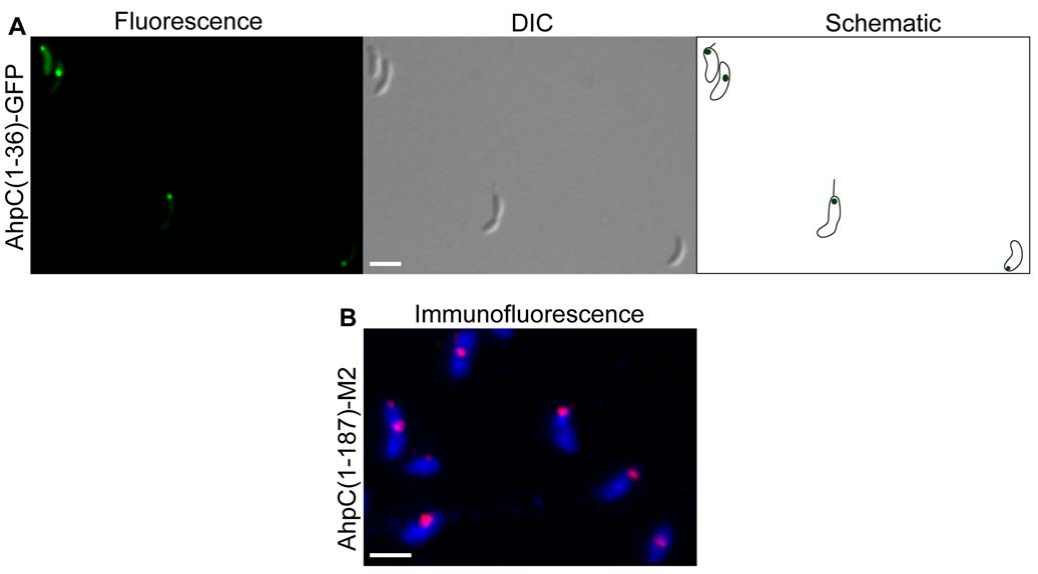
Localization of AhpC. (A) *C. crescentus* producing AhpC(1–36)-GFP generated by insertion of mini-Tn5-GFP were imaged by epifluorescence (left panel) and differential interference contrast (DIC) microscopy (center panel). Schematic diagram (right panel) indicates location of fluorescence signal within the cell. Quantification of images like those shown in panel A indicated 70% of cells had a focus at the stalked pole and 30% had a focus at mid-cell (n = 129). (B) *C. crescentus* producing full-length AhpC fused to M2 were imaged by immunofluorescence microscopy using anti-FLAG M2-Cy3 antibody (fuchsia) and DAPI (blue). Scale bars indicating 1 µm are shown.

Two of the localized GFP fusions were to proteins with known functions but for which no intracellular localization had been reported. AhpC, a subunit of alkyl hydroperoxide reductase, was localized to a single focus at the stalked pole (70%) or mid-cell (30%) when fused to GFP (Table 1, Fig. 2A). AhpC is involved in oxidative stress response, and there is no obvious reason why it would be localized to a subdomain of the cytoplasm. To confirm the epifluorescence results and ensure that truncation of the protein was not responsible for the localization pattern, immunofluorescence microscopy was used to observe the localization of full-length AhpC with an M2 epitope at the C terminus. Immunofluorescence images also showed AhpC in a focus at the stalked pole or mid-cell (Fig. 2B). The second protein, alanine dehydrogenase, was localized in a focus near mid-cell (Table 1). As for AhpC, there is no clear reason why alanine dehydrogenase would be localized. These data indicate that some proteins that do not appear to function in localized reactions or processes are nevertheless concentrated at specific locations in the cell.

**Figure 3:**
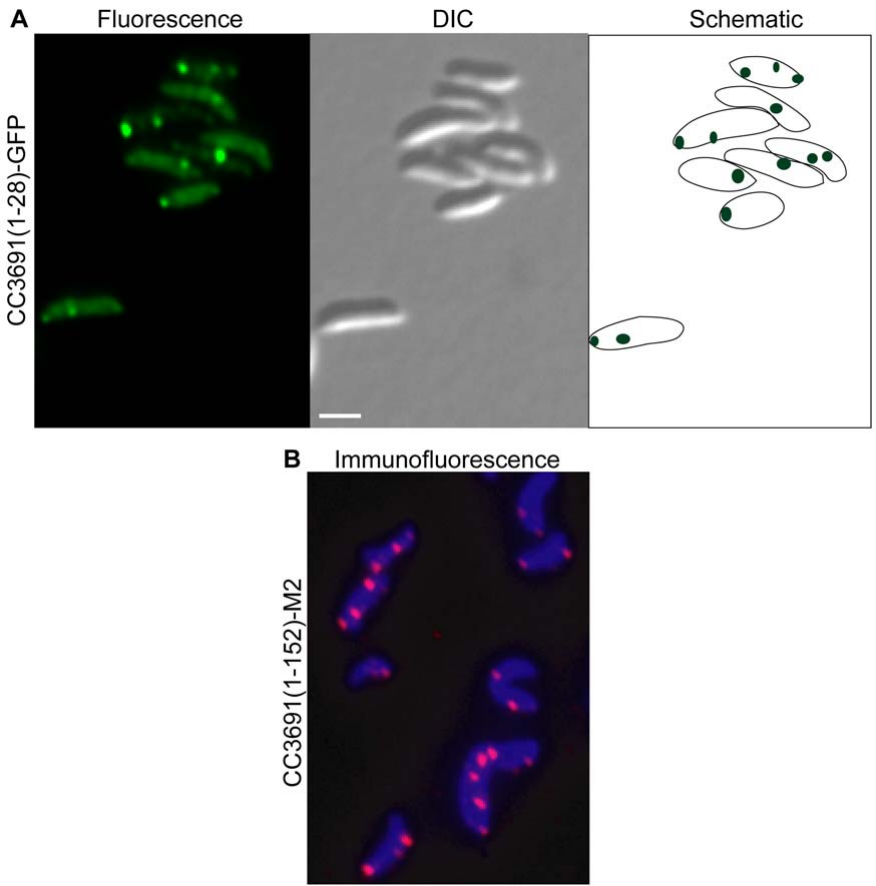
Localization of CC3691. (A) *C. crescentus* producing CC3691(1–28)-GFP generated by insertion of mini-Tn5-GFP were imaged by epifluorescence (left panel) and differential interference contrast (DIC) microscopy (center panel). Schematic diagram (right panel) indicates location of fluorescence signal within the cell. Quantification of images like those shown in panel A indicated 94% of cells had one focus at the stalked pole and one focus at mid-cell (n = 143). (B) *C. crescentus* producing full-length CC3691 fused to M2 were imaged by immunofluorescence microscopy using anti-FLAG M2-Cy3 antibody (fuchsia) and DAPI (blue). Quantification of images like those shown in panel B indicated 98% of cells had one focus at the stalked pole and one focus at mid-cell (n = 80). Scale bars indicating 1 µm are shown.

Four localized GFP fusion proteins were annotated as hypothetical or conserved hypothetical proteins, in addition to CC2362. CC3691-GFP was located in two foci, one at the stalked pole and one at mid-cell in 94% of cells, even though the fusion contains only 28 residues of CC3691 (Table 1, Fig. 3A). Immunofluorescence microscopy of cells producing a full-length CC3691 with an M2 epitope at the C terminus also showed two foci in 98% of cells (Fig. 3B), confirming the localization pattern and indicating that the information directing protein localization is contained within the N-terminal 28 residues. Fusions to CC3385 and CC2233 were localized to the periphery of the cell, indicating that these proteins are associated with the membrane, even though bioinformatic predictions did not suggest membrane interactions. The localization patterns of both CC0572 and CC2233 appeared to depend on the cell type, so these proteins were investigated in more detail.

### CC0572 is localized to a cell cycle-dependent helix-like structure

**Figure 4:**
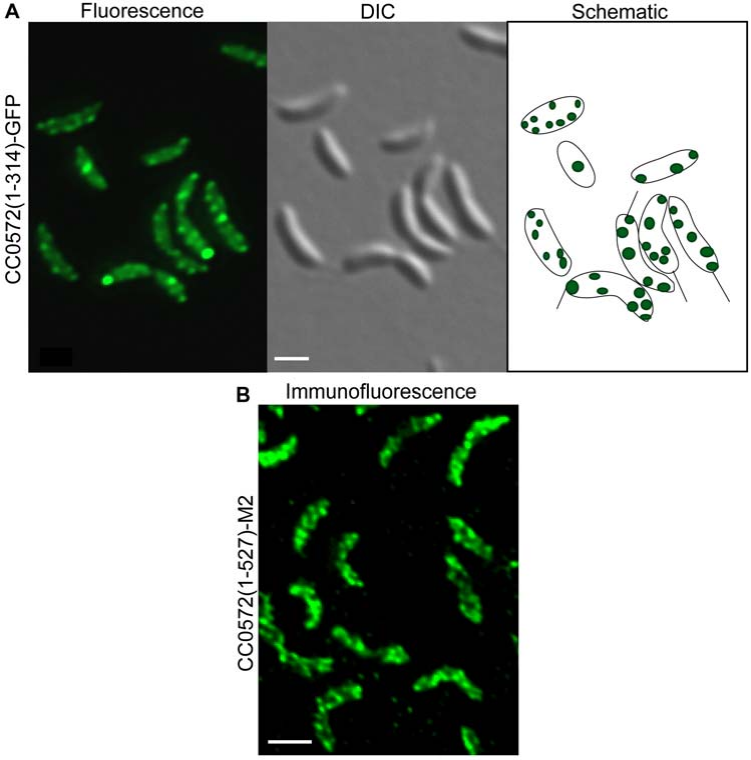
Localization of CC0572. (A) *C. crescentus* producing CC0572(1–314)-GFP generated by insertion of mini-Tn5-GFP were imaged by epifluorescence (left panel) and differential interference contrast (DIC) microscopy (center panel). Schematic diagram (right panel) indicates location of fluorescence signal within the cell. (B) *C. crescentus* producing full-length CC0572 fused to M2 were imaged by immunofluorescence using anti-FLAG antibody and Alexa Fluor 488 anti-mouse antibody (green). Scale bars indicating 1 µm are shown.

**Figure 5:**
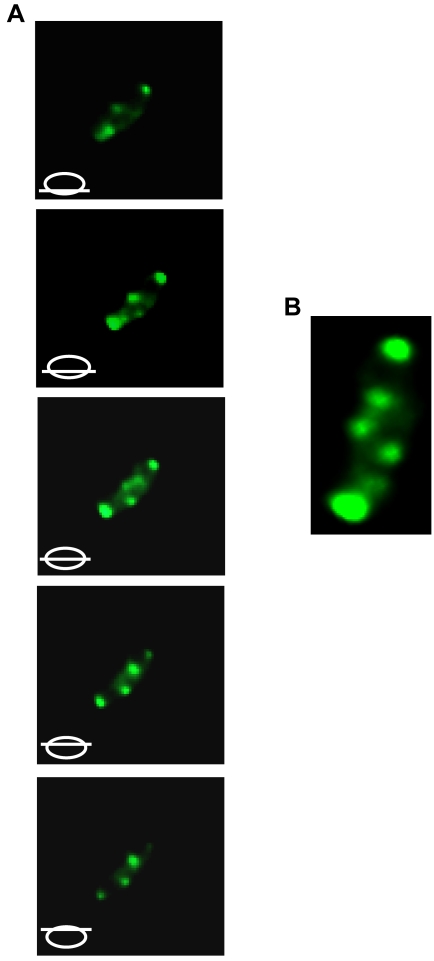
CC0572 is localized to a helix-like structure. (A) Confocal microscopy was used to acquire a Z-series of fluorescence images of *C. crescentus* producing CC0572(1–314)-GFP. Schematic diagrams indicate the relative position of the optical section. (B) Images from the Z-series were computationally reconstituted to produce a composite image.

Epifluorescence images of CC0572 fused to GFP revealed a regular array of spots and bands in over 50% of cells (Fig. 4A). Arrays of spots or bands have been seen in fluorescence images of proteins that form a helix within the cell [8], [27]–[31]. To confirm the localization pattern of CC0572, immunofluorescence images were obtained from cells producing a fusion of the full-length CC0572 protein to M2 (Fig. 4B). As seen for other helical filaments (13, 29), CC0572-M2 produced a pattern of connected bands. To determine if CC0572 was localized in a helix or other three-dimensional structure, optical sections were obtained using confocal microscopy. When these sections were computationally reconstituted to produce a three-dimensional model of the fluorescence signal, a helix-like pattern of CC0572-GFP that extended the length of the cell was apparent (Fig. 5).

**Figure 6:**
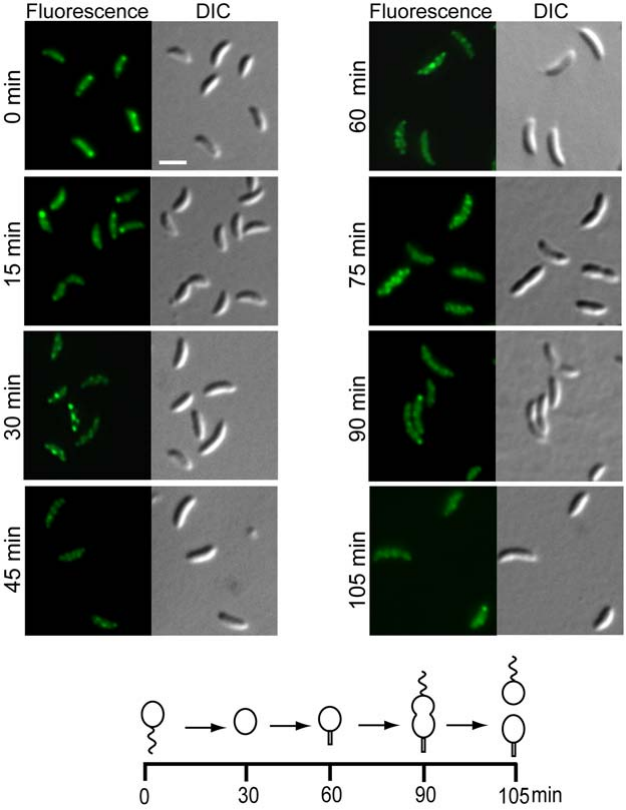
CC0572 localization changes during the cell cycle. Swarmer cells were isolated from cultures of *C. crescentus* producing CC0572(1–314)-GFP and the cells were imaged by epifluorescence (left panels) and DIC microscopy (right panels) during synchronous growth in PYE medium. The time after synchronization is indicated. Schematic diagram indicates the stage of the cell cycle at each time point. Quantification of images like those shown indicated that 43% of cells had a focus at the cell pole at 0 min (n = 105); from 30–75 min a helix-like pattern was seen in 61% of cells (n = 176); and after 90 min, 47% of septating cells retained the helix-like pattern (n = 110). Scale bars indicating 1 µm are shown.

For cells producing CC0572-GFP in exponentially growing cultures, which contained all cell types, only stalked cells clearly had the helix-like fluorescence pattern. To determine if the pattern changed through the cell cycle, swarmer cells producing CC0572-GFP were isolated and the fluorescence was observed as the cells passed synchronously through the cell cycle (Fig. 6). Swarmer cells (0 min) frequently had one bright spot of fluorescence at the cell pole. As the cells differentiated into stalked cells, this spot was lost and the helix-like fluorescence pattern could be seen (30–75 min). In late pre-divisional cells (90 min), the helix-like pattern was maintained, and after cell division, swarmer cells had a single focus of fluorescence and stalked cells had a helix-like fluorescence pattern. Thus, the localization of CC0572 changed as a function of the cell cycle. Western blots showed that the amount of CC0572 protein in the cell changed by less than 20% during the cell cycle (not shown), so the reorganization of the fluorescence signal is unlikely to be due to changes in protein concentration.

**Figure 7:**
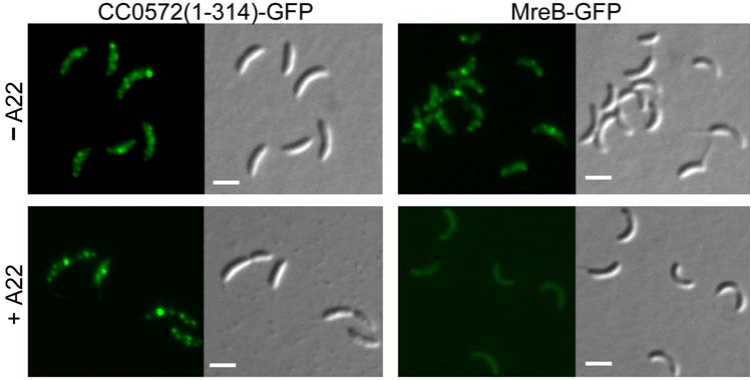
Localization of CC0572 does not depend on the MreB helix. *C. crescentus* producing CC0572(1–314)-GFP or GFP-MreB were incubated without A22 (top) or with A22 (bottom) for 30 min and imaged by epifluorescence (left panels) and DIC microscopy (right panels). Under these conditions, the GFP-MreB helix was completely disrupted, but the CC0572(1–314)-GFP helix was still present. Scale bars indicating 1 µm are shown.

Cell-cycle regulated helical localization patterns have been reported for the actin-like protein MreB in *C. crescentus* [28]. To determine if CC0572 required MreB for localization, the MreB structure was disrupted using the inhibitor A22 [32]. In cells treated with A22, fluorescence from a GFP-MreB fusion was rapidly delocalized (Fig. 7). The helical pattern produced by CC0572-GFP was unaffected under similar conditions (Fig. 7), indicating that localization is independent of the MreB structure.

Localization to a cell-cycle dependent structure suggests a role for CC0572 in a cell-cycle regulated process, but no function for this protein is known. CC0572 has some sequence similarity to chitinases, suggesting that it might be involved in exopolysaccharide metabolism. Consistent with this hypothesis, cells lacking CC0572 could not be infected with phage ΦCr30, which uses the S-layer protein attached to the exopolysaccharide as a receptor [33]. To determine if CC0572 was required for normal cell structure, a deletion of the CC0572 gene was engineered and cell growth and morphology were assessed under a variety of nutrient and growth conditions. No defects were seen for these cells during lag phase, exponential growth, or stationary phase in complex medium or defined media. Likewise, no defects in colony morphology were seen for growth on solid media. These results indicate that although CC0572 is produced and localized during exponential growth in culture, it is not required for most processes under laboratory conditions. CC0572 may play an auxiliary role or provide a redundant enzymatic activity, so that absence of CC0572 is not deleterious. Alternatively, CC0572 may be necessary only under particular growth conditions that were not assayed here.

### CC2233 Moves to the Division Plane During the Cell Cycle

**Figure 8:**
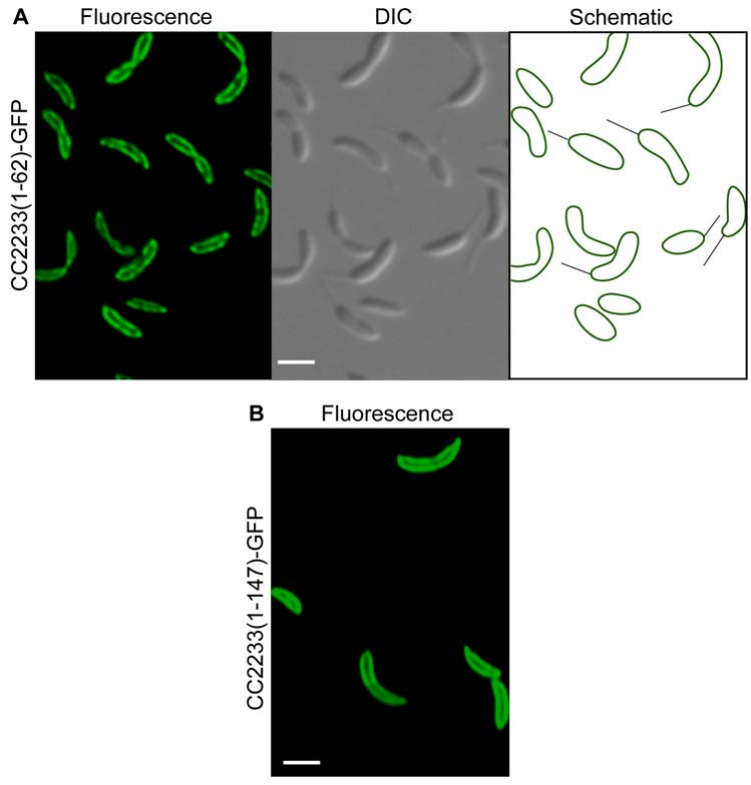
Localization of CC2233. (A) *C. crescentus* producing CC2233(1–62)-GFP generated by insertion of mini-Tn5-GFP were imaged by epifluorescence (left panel) and differential interference contrast (DIC) microscopy (center panel). Schematic diagram (right panel) indicates location of fluorescence signal within the cell. Quantification of images as in (A) indicated 100% of cells had fluorescence localized to the periphery (n = 500). (B) *C. crescentus* producing full-length CC2233 fused to GFP were imaged by epifluorescence. Quantification of images as in (B) indicated 100% of cells had fluorescence localized to the periphery (n = 100). Scale bars indicating 1 µm are shown.

**Figure 9:**
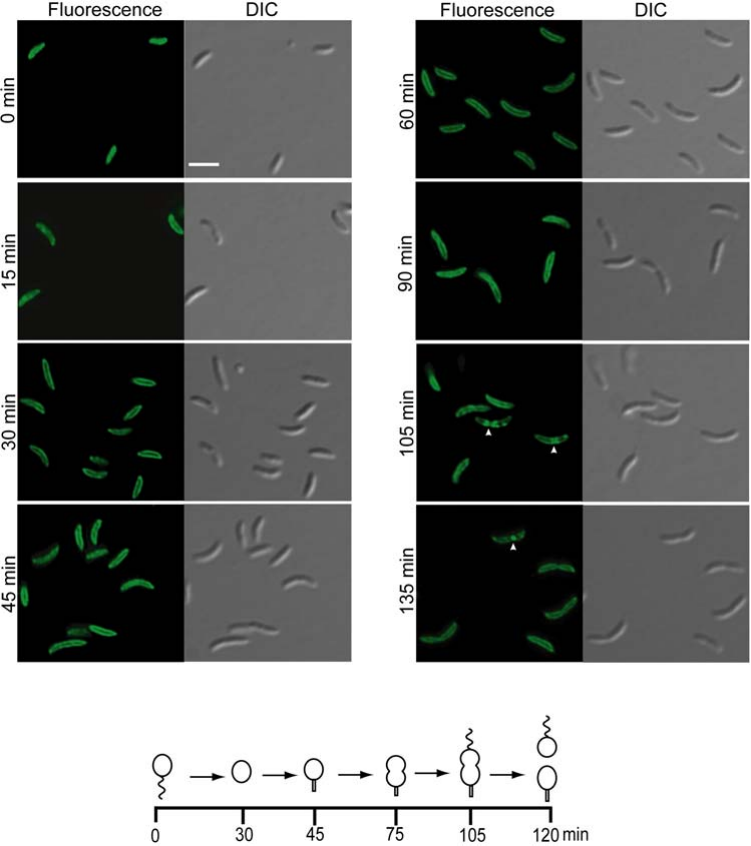
Localization of CC2233 changes during the cell cycle. Swarmer cells were isolated from cultures of *C. crescentus* producing CC2233(1–62)-GFP and the cells were imaged by epifluorescence (left panels) and DIC microscopy (right panels) during synchronous growth in M2G medium. The time after synchronization is shown (cell cycle progression is slower than in Figure 4 because cells were grown in minimal medium). Schematic diagram indicates the cell cycle stage at each time point. CC2233-GFP was located around the periphery until the predivisional stage (105–135 min), when it was concentrated at the division plane (arrowheads). Scale bars indicating 1 µm are shown.

The transposon insertion in *CC2233* resulted in a fusion protein containing only 62 amino acids of CC2233, which was localized to the periphery of the cells in 100% of cells (Fig. 8). Bioinformatic predictions did not suggest that CC2233 is a membrane protein, so to ensure that the full-length protein was also localized, the full-length *CC2233* gene fused to *gfp* was cloned under the control of a vanillate-inducible promoter and expressed in *C. crescentus.* The fluorescence patterns in these cells were indistinguishable from those in the transposon-generated fusion (Fig. 9). Cells producing CC2233-GFP were synchronized and observed to determine if the localization pattern changed as a function of the cell cycle. In swarmer and stalked cells, the fluorescence signal was localized around the periphery of the cell, but in pre-divisional cells the majority of the fluorescence moved to the division plane (Fig. 9). This localization pattern suggests that CC2233 might play some role in the late processes of cell division. However, deletion of CC2233 did not result in any defects in cell division, morphology, or cell growth. It is possible that CC2233 is localized to the division plane through interactions with a cell division protein, but CC2233 itself is not important for cytokinesis. Alternatively, CC2233 may be involved in a non-essential process at the division plane. The function of both CC0572 and CC2233 remain to be established, but their localization patterns suggest that many proteins with complex and dynamic localization patterns remain to be identified.

## Discussion

The transposon-based strategy employed here successfully identified localized proteins in *C. crescentus*, and could be adapted for any species that is compatible with transposon mutagenesis. The first sequencing efforts identified eleven previously unknown localized proteins out of 24 clones that were sequenced. Because over 1000 clones with localized GFP signal were obtained, there are likely to be many more localized proteins in *C. crescentus* than have been described to date. This point is accentuated by two groups of localized proteins, those involved in processes for which there is no obvious requirement for localized function, such as metabolism and stress response, and hypothetical proteins. SerA and alanine dehydrogenase are each localized, even though the substrates and products of amino acid metabolism can presumably diffuse rapidly throughout the cell. SerA is also localized in *E. coli* [9], so it is unlikely that this phenomenon is peculiar to *C. crescentus*. One possible rationale for localizing biosynthetic enzymes is to limit diffusion of unstable or harmful reaction intermediates. Six of the localized proteins were annotated as hypothetical, a category that comprises 37% of the *C. crescentus* proteome [34]. The recovery of these hypothetical proteins at a high frequency from the screen indicates that localization may not be rare. In particular, the identification of two hypothetical proteins with cell-cycle dependent localization patterns, one of which is localized to a helix-like structure, indicate that dynamic localization and complex three-dimensional structures may be common in *C. crescentus*.

For two proteins, the mini-Tn5-GFP screen identified a short peptide that must contain sufficient information to target the protein to its location. The AhpC-GFP fusion contained only 36 residues of AhpC, and CC3691-GFP contained 28 residues of CC3691, yet each of these fusions was localized in the same pattern as the full-length proteins. It has been demonstrated that short peptides, or locons, can target an exogenous protein to a precise location in a bacterial cell [7]. These data indicate that endogenous proteins use locon signals as well.

In principle, proteins in aggregates or inclusion bodies could appear to be localized in one or more foci, and it is possible that some of the clones isolated from the screen have localized GFP signal due to aggregation or inclusion body formation. However, potential aggregation or inclusion body formation due to protein over-production was limited for the screen used here, because proteins were produced from their wild-type promoter at the normal chromosomal locus. In addition, the foci of CheR, AhpC, and CC3691 were not caused by aggregation of truncated GFP-fusion proteins, or by dimerization or aggregation of the GFP sequence, because identical patterns were seen with full-length versions of the proteins fused to the M2 epitope (Figs. 1, 2, and 3).

A mutagenesis-based screen such as the one used here is significantly faster and less expensive than constructing fusions for each gene, and because the same localization patterns were observed for fusions of full-length versions of five of the proteins to M2 or GFP, the results for many proteins would be identical. However, the mutagenesis approach will clearly miss some localized proteins. Because Tn5 clearly has some sequence bias for insertion, the recovery of localized proteins could be expanded by using additional transposons, such as *mariner* and *mu,* engineered to insert *gfp*. Proteins that are essential and cannot tolerate a C-terminal fusion, and proteins that require a free C terminus for localization will not be recovered using transposon mutagenesis. Polar effects caused by transposon insertion may also prevent recovery of some localized proteins. Nevertheless, many new localized proteins can be found with this technique.
